# Enhanced Mechanical, Thermal and Antimicrobial Properties of Additively Manufactured Polylactic Acid with Optimized Nano Silica Content

**DOI:** 10.3390/nano11041012

**Published:** 2021-04-15

**Authors:** Nectarios Vidakis, Markos Petousis, Emanuel Velidakis, Nikolaos Mountakis, Lazaros Tzounis, Marco Liebscher, Sotirios A. Grammatikos

**Affiliations:** 1 Mechanical Engineering Department, Hellenic Mediterranean University, 71410 Heraklion, Greece; vidakis@hmu.gr (N.V.); mvelidakis@hmu.gr (E.V.); mh90@edu.hmu.gr (N.M.); 2 Department of Materials Science and Engineering, University of Ioannina, 45110 Ioannina, Greece; latzounis@uoi.gr; 3 Institute of Construction Materials, Technische Universität Dresden, DE-01062 Dresden, Germany; 4 Department of Manufacturing & Civil Engineering, NTNU-Norwegian University of Science and Technology, Building B’, Teknologivegen 22, 2815 Gjøvik, Norway; sotirios.grammatikos@ntnu.no

**Keywords:** additive manufacturing (AM), three-dimensional (3D) printing, nanocomposites, polylactic acid (PLA), silicone dioxide (SiO_2_), tensile test, flexural test, Charpy’s impact test, Vickers microhardness, scanning electron microscopy (SEM)

## Abstract

The scope of this work was to create, with melt mixing compounding process, novel nanocomposite filaments with enhanced properties that industry can benefit from, using commercially available materials, to enhance the performance of three-dimensional (3D) printed structures fabricated via fused filament fabrication (FFF) process. Silicon Dioxide (SiO_2_) nanoparticles (NPs) were selected as fillers for a polylactic acid (PLA) thermoplastic matrix at various weight % (wt.%) concentrations, namely, 0.5, 1.0, 2.0 and 4.0 wt.%. Tensile, flexural and impact test specimens were 3D printed and tested according to international standards and their Vickers microhardness was also examined. It was proven that SiO_2_ filler enhanced the overall strength at concentrations up to 1 wt.%, compared to pure PLA. Atomic force microscopy (AFM) was employed to investigate the produced nanocomposite extruded filaments roughness. Raman spectroscopy was performed for the 3D printed nanocomposites to verify the polymer nanocomposite structure, while thermogravimetric analysis (TGA) revealed the 3D printed samples’ thermal stability. Scanning electron microscopy (SEM) was carried out for the interlayer fusion and fractography morphological characterization of the specimens. Finally, the antibacterial properties of the produced nanocomposites were investigated with a screening process, to evaluate their performance against *Escherichia coli* (*E. coli*) and *Staphylococcus aureus* (*S. aureus*).

## 1. Introduction

Polylactic acid (PLA) is a commonly used bio-based polymer at a wide range of applications. PLA is produced from agricultural products, such as starch or corn [1] and nowadays is used as a matrix material in combination with diverse nanoparticle fillers for a wider usage intention. PLA exhibits a great behavior when it is used in composites, either as matrix or as a component in a polymer blend system [2]. PLA composites with fillers such as micro-scale fibers, nano- or micro-scaled additives have been suggested for implementation in medical devices [3], in the construction sector [4] and many applications requiring advanced mechanical and thermal properties [5,6,7], or even with specific dielectric behavior, with the addition of appropriate fillers [8].

A wide range of materials have been reported as fillers in a PLA matrix, in order to endow specific properties or to enhance existing ones. Silicon dioxide (SiO_2_), commonly referred as silica, may exist in amorphous and crystalline structure. As quartz-based material it is highly crystalline and can be found as main component of sand [9]. In the construction sector, particularly in concrete industry, crystalline SiO_2_ is widely applied in the shape of aggregates and smaller sized fillers. As amorphous SiO_2_ it is used to accelerate cement hydration [10,11] and tailor 3D printing properties [12].

As a filler in other composites’ development, SiO_2_ is used to achieve specific properties, such as microwave absorption [13]. Silica is also used in coatings [10,14], medicine [15,16], as a reinforcement material [17] and in applications aiming to improve the mechanical response of the polymer matrix [4,18,19]. All these applications and research usages of silica are related to traditional manufacturing processes, such as films production, blend mixing, casting, etc. This work is focused on the study of the properties of PLA/SiO_2_ nanocomposites implemented in additive manufacturing. To the best of the authors’ knowledge, there is no similar research presented in literature, so far.

Additive manufacturing (AM) constitutes an innovative manufacturing technology, gaining a continuously larger share in the manufacturing processes “catalog”. AM meets enough requirements of a sustainable society [20]. This potential of AM process in combination with bio-based materials, such as the PLA, creates a research need for the development of advanced composite materials. PLA has been thoroughly studied and it is continually under development with a large variety of fillers [2,8,21] and applications related to 3D printing for the development of composites with antimicrobial properties [22]. Polylactide is a famous polymer used in 3D printing, with an “easy-to-print” behavior. It is also referred that it has a much better processability for filament fabrication, when used in the development of nanocomposite materials [23,24,25].

In this study, PLA was mixed with silicone dioxide in different filler percentages in order to produce PLA/SiO_2_ nanocomposite filaments. The filaments were purposed to be used in 3D printing to manufacture all needed specimens for a wide variety of mechanical, morphological, thermal and antibacterial tests. The only research presented in the literature so far with the same materials combination in 3D printing is the work by Siraj et al., in which recycled PLA was mixed with sand (which consists of crystalline silica in most) [4]. This work has many limitations and focuses on the study of different properties, compared to the current work, since it focuses and reports on only the tensile properties of the 3D printed specimens.

In the study at hand, nano-silicon dioxide is added as a filler in PLA in low percentages and specimens tested are manufactured through 3D printing, as the processability of the filament should have been also checked. Static loading mechanical tests were implemented, i.e., tension, flexion, impact and microhardness, while, additionally, various thermal and morphological studies were conducted (Thermogravimetric Analysis—TGA, Raman, Scanning Electron Microscopy—SEM, Atomic force microscopy—AFM), to further analyze the effect of the filler in the polymer matrix. It was found that the PLA/SiO_2_ with 1 wt.% had the highest mechanical performance among the four concentrations studied. Finally, the antimicrobial properties of the 3D printed nanocomposites were examined, and a mild antibacterial response was found in the nanocomposites with high filler loadings (4.0 wt.%).

## 2. Materials and Methods

### 2.1. Materials

Polylactic acid (PLA), chosen for this study as a matrix material, is of 3052D grade, with molecular weight 116,000 g/mol, procured from Plastika Kritis SA (Heraklion, Greece) in form of coarse powder. Silicon dioxide (SiO_2_) was chosen as a filler material at four (4) different mixing ratios. Silicon dioxide procured in nano-powder form of 5–15 nm grain size from Sigma-Aldrich Chemie GmbH (Taufkirchen, Germany). Silicon dioxide nanoparticles concentration in powder was >99.5% and nanoparticles are of spherical shape.

### 2.2. Methods

**Figure 1:**
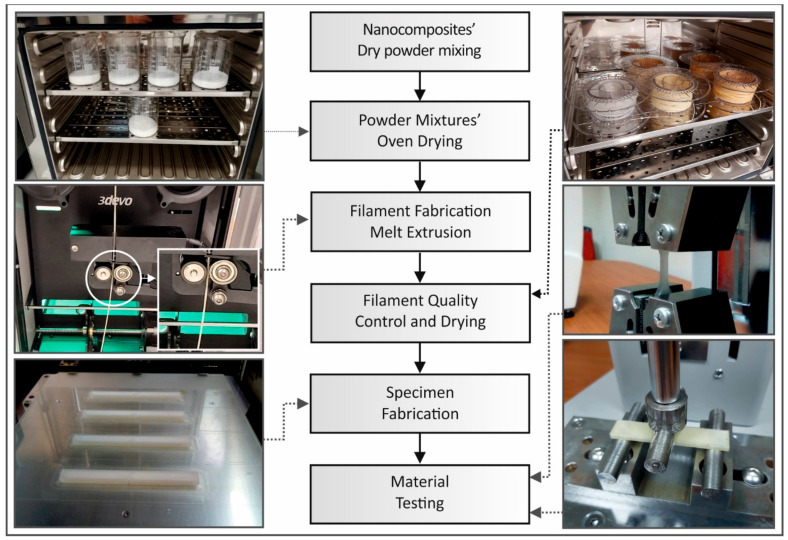
Methodology flow chart followed in this study, in a combination with pictures captured during test procedures.

The methodology followed in this work is illustrated and summarized in Figure 1.

#### 2.2.1. Filament Fabrication

Matrix material (PLA) and filler (silicon dioxide) were chosen to be tested under four (4) different mixing ratios. Mixing percentages were of 0.5%, 1.0%, 2.0% and 4.0% in a weight to weight (*w*/*w*) approach. Pure PLA had also been produced as a reference to all composite materials. Adequate quantity of each material was weighted and mixed using mechanical mixing equipment. In order to ensure the lack of humidity in all materials developed in the study, drying procedures were used in all steps of the process. PLA coarse powder was dried for 24 h at 50 °C using a laboratory oven. Further drying followed the mixing process for all materials, in this case for 5 h at 50 °C. 1.75 mm in diameter filament suitable for use in FFF 3D printing was produced, with a melt mixing thermomechanical process, using a 3D Evo (3D Evo B.V., NL) single screw extruder.

Specifically, 3D Evo Composer 450 model was employed for the extrusion procedure. This extruder uses an optimized for mixing screw. Its chamber has four heating zones, and the device features also a filament diameter real-time measurement system. In this way, produced filament is undergoing quality control during the production process, for the diameter dimensions accuracy and the extrusion parameters are automatically adjusted accordingly, to maintain as good as possible the prescribed accuracy tolerances.

A 1.75 mm diameter filament was produced for all material concentrations, while the mean diameter deviation was calculated to be 0.06 mm. All materials produced, i.e., pure PLA and the four nanocomposites produced in this study, were processed under same extrusion parameters. Temperatures were set to each heating zone; 175 °C at heat zone 4 (closer to hopper), 205 °C at heat zone 3 and 2 (middle stage) and 195 °C at heat zone 1 (closer to extruder’s nozzle). Screw rotational speed was set to 7.4 rpm and the built-in winder was automatically set to rotational speeds in order to achieve the requested diameter. Additional quality control tests were also manually conducted with random diameter measurements to the filament diameter using a caliper and optical quality control.

#### 2.2.2. Tensile Specimens’ Fabrication and Testing

**Figure 2:**
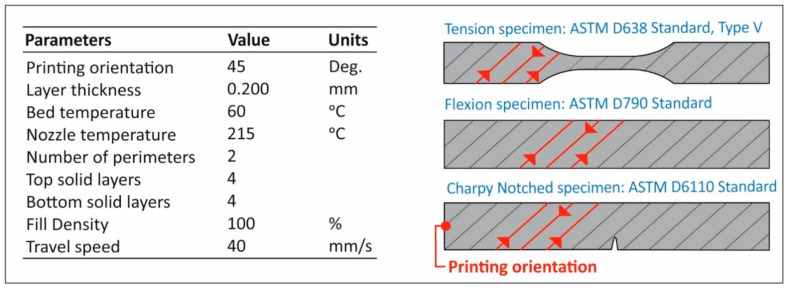
3D Printer’s fundamental parameters set up to slicer software.

Specimens were manufactured using fused filament fabrication (FFF) AM technology. An Intamsys (Shanghai, CN) 3D Printer was chosen using an extruder’s nozzle of 0.4 mm. Specifically, Intamsys HT 3D printer was used for specimens’ fabrication through AM. In Figure 2, the complete list of the 3D printing parameters used in the study for the fabrication of the specimens in the 3D printer, is shown. All other parameters were set to default in the Intamsuite software tool (choosing PLA as the reference material in the 3D printer software tool), which was used as the slicer tool for this study.

American Society for Testing and Materials (ASTM) D638-02a international standard was followed in the tensile testing procedure. According to the standard, a type V specimen of 3.2 mm thickness was chosen, and five (5) specimens were 3D printed and tested for each case. Tensile tests were conducted on an Imada MX2 (Imada Inc., Northbrook, IL, USA) quasi-static testing apparatus, at a crosshead speed of 10 mm/min and room temperature conditions (~22 °C and 50%RH).

#### 2.2.3. Flexure Specimens Fabrication and Testing

Flexural test specimens were fabricated using AM technology. 3D printing parameters were the same as these referred above (tensile specimens’ fabrication). Flexure tests were conducted according to the ASTM D790-10. Five (5) specimens were manufactured at a 3.2 mm thickness and tested using the same apparatus referred above, employing a three-point bending flexural mode setup. Apparatus crosshead speed was set to 10 mm/min and all tests were conducted at room temperature conditions (~22 °C and 50%RH).

#### 2.2.4. Impact Specimens Fabrication and Testing

Identical parameters and equipment were used for the 3D printing of the impact specimens. Impact tests were conducted according to the ASTM D6110-04. Specimens’ dimensions were 80 mm length, 8 mm width and 10 mm thickness. A total of five (5) specimens were tested in a Charpy impact test apparatus. The apparatus employed for the tests was a Terco MT 220 Charpy (Terco AB, Kungens Kurva, Sweden). The release height of apparatus hammer was the same for all tests, which were conducted at room temperature conditions (~22 °C and 50%RH).

#### 2.2.5. Micro-Hardness Measurements

Vickers-Microhardness measurements were conducted according to the ASTM E384-17. Measurements were taken on randomly selected specimens, from tensile, flexure and impact specimens. All specimens were thoroughly prepared according to standard’s requirements. An Innova Test 300-Vickers (Innovatest Europe BV, Maastricht, The Netherlands) was used for the measurements. The applied force was set to 200 grF and indentation’s duration was 10 s. Imprints were measured under five (5) different specimens for each material. All tests were conducted at room temperature conditions (~22 °C and 50%RH).

#### 2.2.6. Antibacterial Measurements and Specimens’ Fabrication

To determine the antibacterial properties of the nanocomposite materials developed in this work, the agar well diffusion method [26] was employed and implemented in a microbiological lab, for two different bacteria, i.e., *Escherichia coli (E. coli)* and *Staphylococcus aureus (S. aureus)*. 850 mm in diameter Petri dishes with suitable for each bacterium, bacterium growth material, were employed for the tests. Two cylindrical specimens (one for each bacterium) of 12.00 mm diameter and 5.00 mm height were 3D printed with the same parameters described above, from pure PLA and each one of the four different nanocomposite material prepared in this work, so a total of ten specimens were fabricated and tested.

The bacteria were inoculated in the corresponding Petri dishes and the specimens were also placed in the Petri dishes near their center. One specimen was placed in each Petri dish. The Petri dishes were placed in an oven at 37 °C for 24 h in order for the antimicrobial agent of the nanocomposite to diffuse into the agar and inhibit germination and growth of the test microorganism. Then, the diameters of the inhibition growth zones were measured.

#### 2.2.7. Characterization Techniques

Raman spectroscopy was performed with a Labram HR-Horiba (Horiba Scientific, Kyoto, Japan) scientific micro-Raman system. All spectra were acquired in the back-scattering geometry with a 514.5 nm line of an Ar^+^ ion laser operating at 1.5 mW power at the focal plane. In order to facilitate the excitation light onto the sample’s surface as well as collecting the back-scattering Raman activity, a 50× long working distance objective has been utilized as part of an optical microscope set-up.

Thermogravimetric analysis (TGA) was carried out for the pure PLA as well as the PLA/SiO_2_ nanocomposites using a NETZSCH STA 409C/CD instrument (NETZSCH Gerätebau GmbH, Selb, Germany). The TGA runs have been conducted in oxygen atmosphere from ambient (25 °C) up to 800 °C at a heating rate of 10 K/min, while Curie point standards were utilized for the temperature calibration. TGA scans, as well as the first derivative, known as differential thermogravimetry (DTG) analysis, have been shown for the temperature window of 70–450 °C.

Scanning electron microscopy (SEM) investigations were performed using an FEI NanoSem 200 (FEI, Eindhoven, The Netherlands) at an accelerating voltage between 1–2 kV. The analysis was run on fractured surfaces as well as non-tested areas of 3D printed layers. To avoid loss of information, no sputter coating was applied on the non-conductive samples.

## 3. Results

### 3.1. Tensile Results

**Figure 3:**
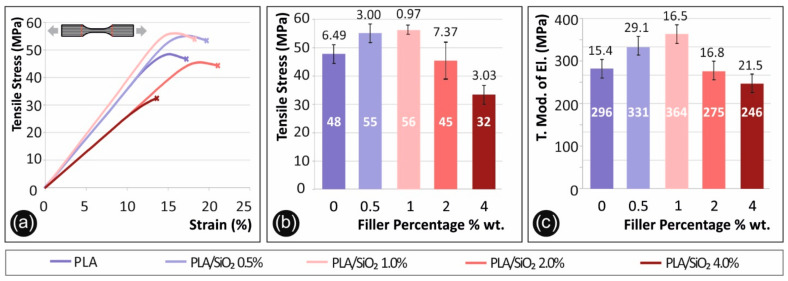
Tensile test results, (**a**) tensile stress (MPa) to strain (%) graph of a representative specimen of each material tested, (**b**) mean max measured tensile stress (at break) (MPa) and standard deviation to filler percentage (%) (0.5% wt. developed 13.8% increased values, when compared to pure Polylactic acid (PLA), 1% wt. 15.6% increase, 2% wt. 6.0% decrease and 4% wt. 33.0% decrease, respectively), (**c**) mean tensile modulus of elasticity (MPa) to filler percentage (%) (0.5% wt. developed 12.0% increased values, when compared to pure PLA, 1% wt. 23.1% increase, 2% wt. 7.2% decrease and 4% wt. 16.9% decrease, respectively).

Figure 3 below shows results regarding tensile tests conducted according to specifications described above. In Figure 3a, tensile stress (MPa) to calculated strain (%) graph of a typical specimen tested from each nanocomposite material prepared in this work, in comparison to the pure PLA polymer, is presented. In Figure 3b, the average tensile strength at break (MPa) for each material studied is shown in correspondence to the filler percentage, while in Figure 3c, the average calculated tensile modulus of elasticity (MPa) of each filler percentage tested is shown in comparison to the pure PLA polymer.

### 3.2. Flexural Results

**Figure 4:**
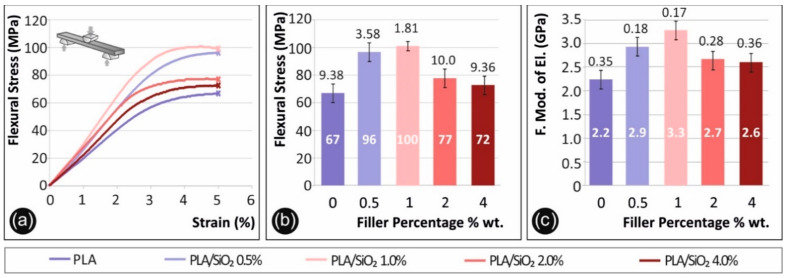
Flexural test results, (**a**) flexural stress (MPa) to strain (%) graph of a representative specimen of each material tested, (**b**) mean max measured flexural stress (at break) (MPa) to filler percentage (%) (0.5% wt. developed 44.1% increased values, when compared to pure PLA, 1% wt. 51.0% increase, 2% wt. 15.9% increase and 4% wt. 8.7% increase, respectively), (**c**) mean flexural modulus of elasticity (MPa) to filler percentage (%) (0.5% wt. developed 30.9% increased values, when compared to pure PLA, 1% wt. 46.6% increase, 2% wt. 19.3% increase and 4% wt. 16.4% increase, respectively).

Figure 4 below presents results from the flexural tests. Flexural stress (MPa) to strain (%) graphs of a typical specimen tested from each nanocomposite material prepared in this work in comparison to the pure PLA polymer, are presented in Figure 4a. Curves in Figure 4a have been chosen as representative stress to strain curves after the average values were calculated for each material. Figure 4b presents the flexural strength (MPa) to filler percentage (%) used in each nanocomposite prepared in the study, in comparison to the pure PLA polymer. Flexural strength average values were calculated at max strain of (5%), according to the ASTM D790-10 standard, as no break occurred on the specimens during testing. In Figure 4c, the average calculated flexural modulus of elasticity (MPa) of each filler percentage tested is shown in comparison to the pure PLA polymer.

### 3.3. Impact-Microhardness Results

**Figure 5:**
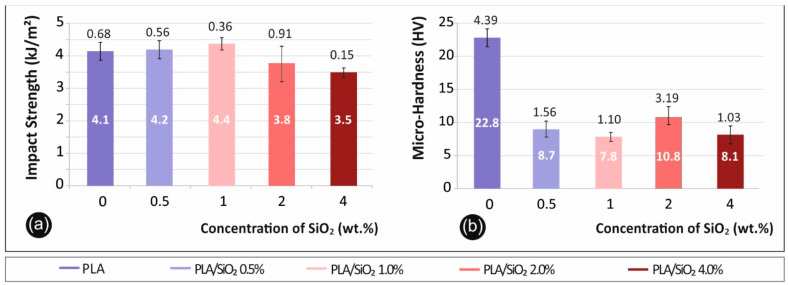
(**a**) Impact strength (kJ/m^2^) to filler percentage (%) in PLA (0.5% wt. developed 0.6% increased values, when compared to pure PLA, 1% wt. 5.4% increase, 2% wt. 8.7% decrease and 4% wt. 15.8% decrease, respectively) (**b**) Vickers microhardness (HV) to filler percentage (%) (0.5% wt. developed 63.7% decreased values, when compared to pure PLA, 1% wt. 65.7% decrease, 2% wt. 52.4% decrease and 4% wt. 64.4% decrease, respectively).

Charpy notched impact test results are shown in Figure 5a. Specifically, in Figure 5a the average calculated impact strength (kJ/m^2^) to filler percentage (%) of each material tested is presented. Figure 5b presents the average micro-hardness Vickers (HV) values calculated after measurements for each material in correspondence to the filler percentage (%).

### 3.4. Antibacterial Results

**Figure 6:**
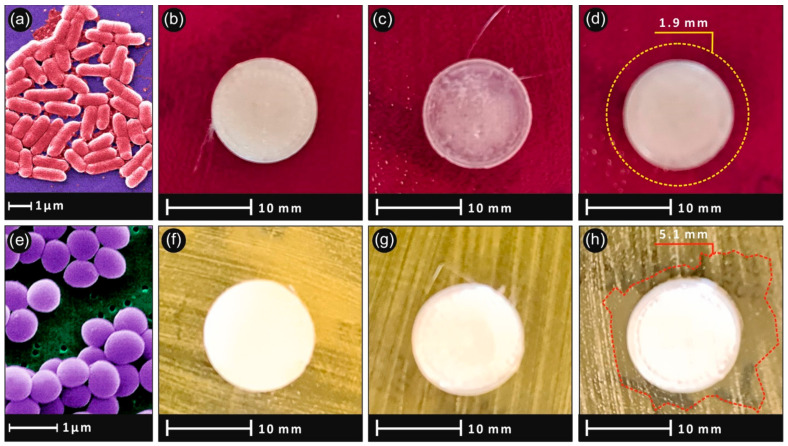
Agar well diffusion method pictures after 24 h culture showing the inhibition growth of *Escherichia Coli* bacteria (**a**) of (**b**) PLA/SiO_2_ (1.0 wt.%), (**c**) PLA/SiO_2_ (2.0 wt.%), (**d**) PLA/SiO_2_ (4.0 wt.%) and *Staphylococcus aureus* (**e**) for (**f**) PLA/SiO_2_ (1.0 wt.%), (**g**) PLA/SiO_2_ (2.0 wt.%), (**h**) PLA/SiO_2_ (4.0 wt.%).

Figure 6 shows the antibacterial results after the 24 h bacteria culture. As expected, no inhibition zone was developed in pure PLA specimens. For the nanocomposites, a mild clearly visible antibacterial action was observed, for the highest concentration samples. The developed inhibition zone was wider in the *Staphylococcus aureus* (Figure 6h) and narrower in the *Escherichia coli* bacteria (Figure 6d), while no inhibition zone was observed in lower filler concentrations.

### 3.5. Raman Spectra of 3D Pritned PLA and PLA/SiO_2_ Nanocomposites

**Figure 7:**
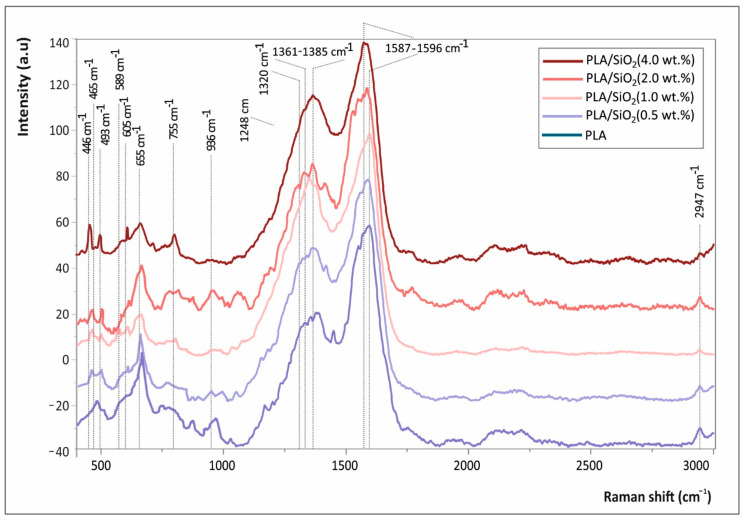
Raman spectra of pure PLA, as well as PLA/SiO_2_ nanocomposites at 0.5, 1.0, 2.0 and 4.0 wt.% fractions.

Figure 7 shows the Raman spectra of pure PLA, as well as PLA/SiO_2_ nanocomposites.

### 3.6. Thermogravimetric Analysis of 3D Printed Neat PLA and PLA/SiO_2_ Nanocomposites

**Figure 8:**
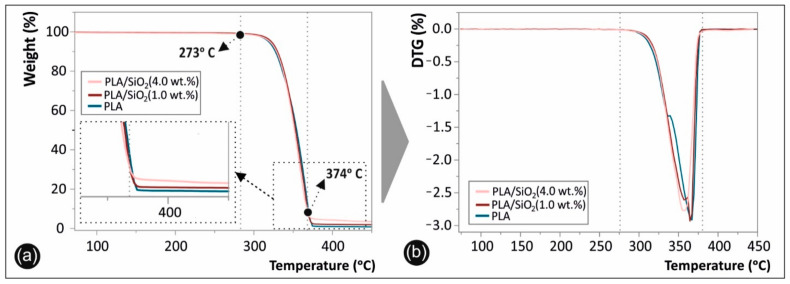
(**a**) Thermogravimetric analysis (TGA) and (**b**) Differential thermogravimetry (DTG) plots of 3D printed neat PLA and PLA/SiO_2_ nanocomposites at 1.0 and 4.0 wt.% filler loading (the inset in Figure 8a shows more specifically the materials weight loss as remnant from 374 °C that all polymeric substance has been decomposed and up to 450 °C, corresponding to the SiO_2_ NPs).

Figure 8 depicts the TGA (Figure 8a), as well as the DTG (Figure 8b) graphs of the 3D printed neat PLA and PLA/SiO_2_ nanocomposites (representative only the 1.0 and 4.0 wt.% filler loading nanocomposites have been tested and shown).

### 3.7. SEM Microstructural Analysis

**Figure 9:**
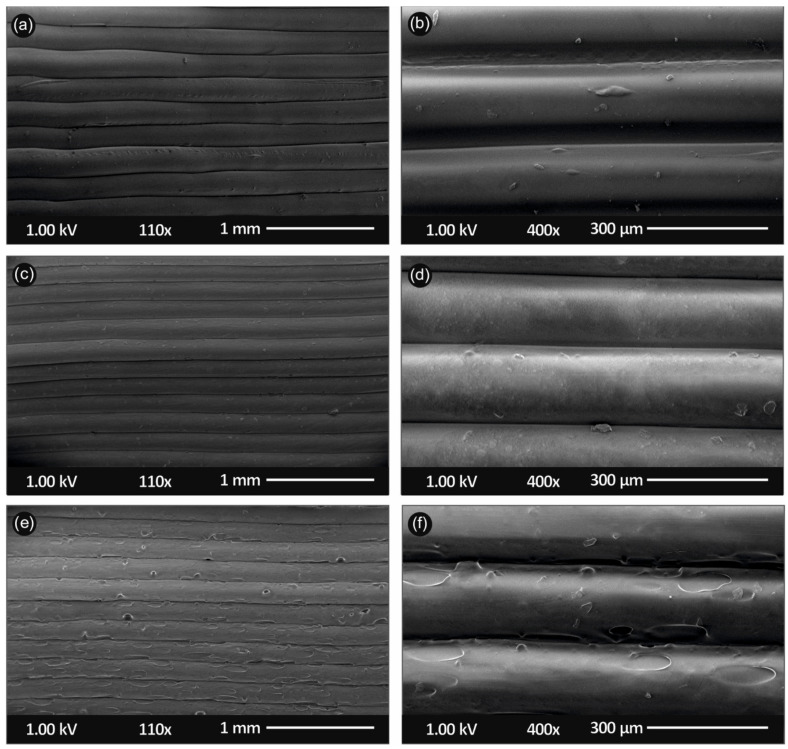
Scanning electron microscopy (SEM) images of the side-surface microstructure and morphology for the different 3D printed samples in this study at two different magnifications (all 3D printed samples with 300 µm 3D printed layer thickness): SEM images at 110× and 400× magnification for the (**a**,**b**) pure PLA, (**c**,**d**) PLA/SiO_2_ (1.0 wt.%) and (**e**,**f**) PLA/SiO_2_ (4.0 wt.%) nanocomposites, respectively.

Figure 9 shows the side surface morphology of the 3D printed PLA, as well as PLA/SiO_2_ nanocomposites (1.0 and 4.0 wt.%) at two different magnifications, illustrating the microstructural characteristics of the samples arising from the additive manufacturing process.

**Figure 10:**
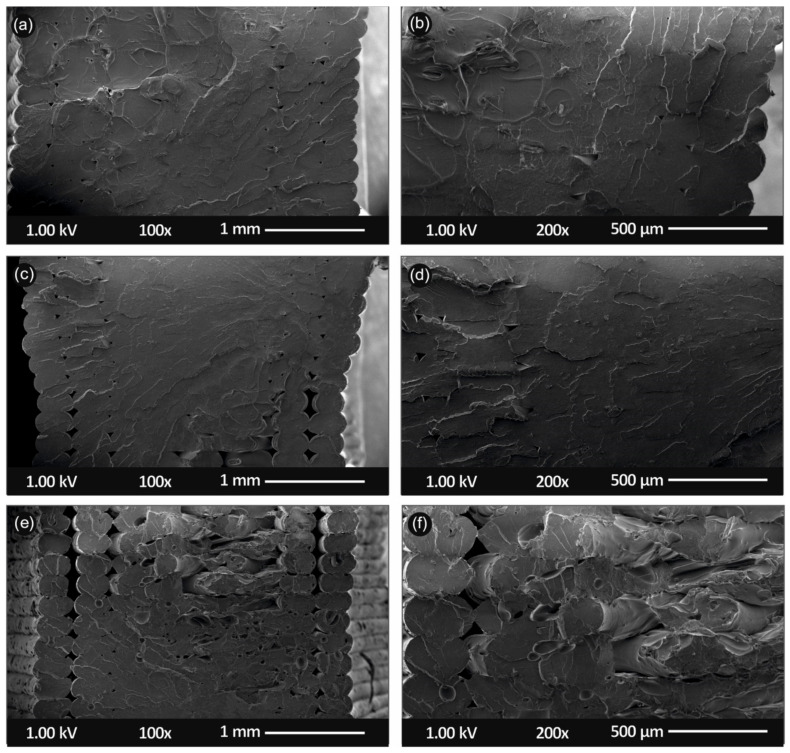
Scanning electron microscopy (SEM) images of the tensile test fractured areas microstructure and morphology for the different 3D printed samples in this study at two different magnifications (all 3D printed samples with 300 µm 3D printed layer thickness): SEM images at 100× and 200× magnification for the (**a**,**b**) pure PLA, (**c,d**) PLA/SiO_2_ (1.0 wt.%) and (**e**,**f**) PLA/SiO_2_ (4.0 wt.%) nanocomposites, respectively.

Figure 10 shows SEM images of the tensile test fractured areas at two different magnifications of the neat PLA specimens (Figure 10a,b), as well as PLA/SiO_2_ (1.0 wt.%) (Figure 10c,d) and PLA/SiO_2_ (4.0 wt.%) (Figure 10e,f) nanocomposites, respectively.

### 3.8. AFM Filament Measurements

**Figure 11:**
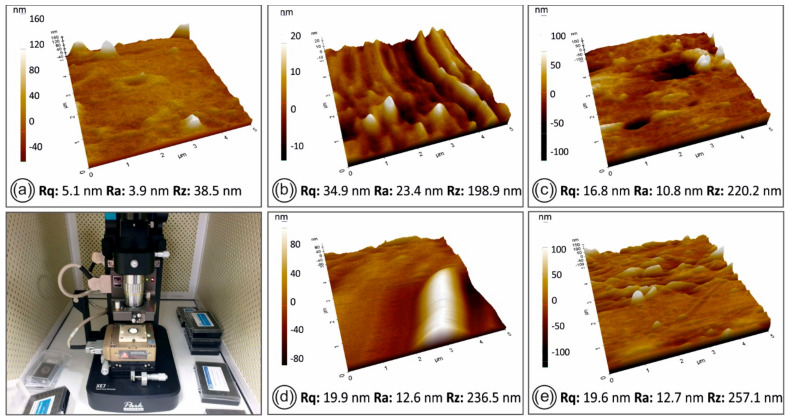
AFM images of the filament side surface for the different materials in this study: (**a**) pure PLA, (**b**) PLA/SiO_2_ (0.5 wt.%), (**c**) PLA/SiO_2_ (1.0 wt.%), (**d**) PLA/SiO_2_ (2.0 wt.%), (**e**) PLA/SiO_2_ (4.0 wt.%) nanocomposites, respectively. On the bottom left picture, the experimental setup for the AFM measurements is shown.

The AFM surface topography and roughness measurements of the filaments, namely pure PLA and the four nanocomposites developed in this study, are shown in Figure 11.

## 4. Discussion

### 4.1. Mechanical Properties

As shown in Figure 4, the tensile strength is increasing accordingly to filler percentage until 1.0 wt.%, where it reaches its highest value. Specifically, an increase of approximately 16.7 wt.% was observed to the tensile stress at break. It was revealed that when silica is at 4.0 wt.%, the tensile strength decreases almost 50% when compared to the highest measured value. It should also be mentioned that the addition of silicon dioxide in the PLA matrix makes the polymer more brittle during the tensile test.

A similar behavior was observed also for the flexural test results. The specimens did not fail up to 5% strain, in which the experiment was terminated as the standard instructs. Again, the highest flexural stress was measured at samples with 1.0 wt.% silica loading, representing an increase of almost 50% in comparison to the un-modified (reference) sample case.

Impact strength showed a lower dependency on filler loading, still a slight increase in impact strength with 1.0 wt.% of nano silica compared to pure PLA samples case was observed. Micro-hardness results exhibited instead a rather stable behavior when silicone dioxide is added to PLA. However, the overall measured micro-hardness is significantly lower in the nanocomposites, when compared to pure PLA, but the filler fraction seems to have negligible effect on the nanocomposites’ microhardness measurements.

Overall, the introduction of silicon dioxide nanoparticles renders the PLA matrix more brittle, while an increase in the tensile and the flexural strength is observed. Similar tests in specimens built with additive manufacturing have not been presented yet to the literature, although similar studies with silica nanoparticles and PLA polymer matrix have been introduced with other processes and material grades. Wu J et al. [27] and Lv H et al. [28] conducted similar research and their results show a similar trend with the results of the current study.

### 4.2. Antibacterial Properties

The antibacterial results presented in Figure 6 show that the addition of SiO_2_ in the PLA matrix can introduce antibacterial properties to the polymer, for concentrations of 4 wt.%, at least for the two bacteria strains tested in this study. This is an important finding, considering that the developed nanocomposite was 3D printed, so it is possible to build parts with this specific process for various types of applications, since FFF 3D printing is such a popular process nowadays with a great potential in personalized biomedical objects/parts.

On the other hand, the mechanical tests showed that increasing the filler percentage more than 1 wt.%, has a negative effect on the mechanical properties of the nanocomposite, with the experimental data showing a descending trend. The reduction is not significant, considering that the 4 wt.% has similar flexural strength with the pure PLA and additionally it has antibacterial properties. Still, this has to be considered in higher concentrations, when designing parts that require antimicrobial properties to be 3D printed with this nanocomposite.

### 4.3. Raman Spectra of 3D Pritned PLA and PLA/SiO_2_ Nanocomposites

All peaks shown in Figure 7 are attributed to the PLA macromolecular chains’ chemistry (backbone chain, side and end-terminal groups) are depicted with continuous lines, while the specific bands assigned to SiO_2_ NPs are illustrated with dashed lines. In specific, both spectra show the characteristic fingerprints of PLA presenting peaks at 465 and 589 cm^−1^ (C-O-C vibration), 655 cm^−1^ (C=O stretching vibration), 936 cm^−1^ (C-COO vibration), 1249 and 1320 cm^−1^ (CH deformation vibration), 1361–1385 cm^−1^ (CH_3_ deformation vibrations of PLA), 1587–1596 cm^−1^ (asymmetric C=O stretching vibrations of carboxylate groups of PLA) and 2947 cm^−1^ (CH_3_ symmetric and asymmetric stretching vibration) [29]. The spectra of PLA/SiO_2_ nanocomposites at the different SiO_2_ wt.% loadings exhibit some peaks attributed to the SiO_2_ NPs vibrational models. Namely, the peaks that appear due to the SiO_2_ NPs blended in the PLA matrix are at ca. 446 cm^−1^, 493 cm^−1^, 605 cm^−1^ and 795 cm^−1^, being in good agreement with the typical SiO_2_ NP vibrational modes reported elsewhere [30]. It is worth mentioning and it can be observed that the SiO_2_ NP peaks’ intensity is slightly increasing with the increased SiO_2_ wt.% fraction.

### 4.4. Thermogravimetric Analysis of 3D Printed Neat PLA and PLA/SiO_2_ Nanocomposites

The TGA and the corresponding DTG plots are presented in Figure 8 in the temperature window of 70–450 °C, while it should be mentioned that all samples have been manufactured following the same process parameters i.e., extrusion parameters for preparing the 3D printing filament as well as the 3D printing FFF process conditions. Namely, three distinct thermal windows can be observed. The first one is up to 273 °C that all materials exhibited thermal stability without any observed weight loss (%). It can therefore be deduced that all PLA and PLA/SiO_2_ nanocomposites are stable up to ca. 270 °C. From 273 °C up to 374 °C, there is a second temperature window, corresponding to the materials’ thermal degradation and decomposition with the onset temperature of decomposition (T_on_) at 273 °C.

All the polymeric substance has been decomposed up to 374 °C, while the remaining weight loss observed for the PLA/SiO_2_ (1.0 wt.%) and PLA/SiO_2_ (4.0 wt.%) from 374 °C up to 450 °C temperature window, corresponds to the 1.0 wt.% and 4.0 wt.% SiO_2_ NP filler loading, respectively. In Figure 8a, the inset shows, more specifically, the material’s weight loss, as well as the remnant from 374 °C and that all polymeric substance has been decomposed and up to 450 °C, corresponding to the SiO_2_ NPs (known to be thermally stable even at temperatures > 1000 °C). It is worth mentioning that all materials exhibited very similar decomposition characteristics, especially for the behavior of the organic/polymer substance used as the matrix, corroborating the extremely high control of the filament extrusion and 3D printing process applying the same parameters and resulting, thus, in the same thermal treatment history for all the manufactured samples. Moreover, the mass loss for the nanocomposite sample, having 4 wt.% SiO_2_ NPs, is lower compared to the 1 wt.% SiO_2_ and pure PLA, due to the fact that only the organic part is degraded. In addition, the overall thermal stability of the samples does not seem to be impacted by the presence of the SiO_2_ filler. The SiO_2_ nanoparticles blended within the PLA matrix at the different loadings in this study, have an average size in the range of 20 nm and are considered much bigger compared to the proteins that have been found to be released as reported elsewhere [31].

Moreover, the SiO_2_ nanoparticles which exhibit the well-known silanol groups (Si-OH) are expected to have strong interactions with the PLA macromolecular chains via H-bond formation, not allowing their release to the environment. A possible release could occur maybe in the case that the PLA/SiO_2_ nanoparticles will be placed in a specific environment, i.e., acidic water, etc., in which the polymer could start to swell. Even if such experiments would be of high interest, this is not the focus of the study at hand, which is mainly focusing on the mechanical properties of the 3D printed manufactured specimens, as well as the examination of their antibacterial properties.

### 4.5. SEM Microstructural Analysis

The 3D printed sample’s microstructure, investigated from the 3D printed side surface (Figure 9), could reveal the 3D printed layer thickness (all samples were 3D printed with 300 µm layer thickness), as well as the quality of printing, showing the sample layers interface characteristics and interlayer fusion. Figure 9a,b show the side-surface morphology of the pure PLA 3D printed sample, while Figure 9c,d for the PLA/SiO_2_ (1.0 wt.%) and Figure 9e,f for the PLA/SiO_2_ (4.0 wt.%) nanocomposites, respectively.

For all samples, a homogeneity in the 3D printing layer thickness can be observed with a layer thickness of approximately 300 μm, in a good agreement with the resolution of the Intamsys Funmat HT 3D printer manufacturer’s technical specifications in terms of 3D printer’s resolution. Moreover, a relatively high quality of bonding/interlayer fusion can be observed, indicating (i) the optimum set of the selected printing parameters, as well as (ii) the high quality of the produced PLA/SiO_2_ NP nanocomposite filaments, i.e., dispersion state of the SiO_2_ NPs in the PLA matrix, structural homogeneity and diameter uniformity produced in our study by melt-mixing process.

It is also worth mentioning that it can be observed by the SEM images that high quality 3D printed objects have been manufactured with good adhesion between the sequencing layers, which could most likely positively affect the mechanical properties of the 3D printed samples. Finally, only in the case of PLA/SiO_2_ (4.0 wt.%) 3D printed sample’s side-surface morphology, few structural defects could be observed, possibly attributed to some slight SiO_2_ NP aggregation and/or the higher viscosity of the PLA/SiO_2_ (4.0 wt.%) melt, due to the high amount of SiO_2_ NPs.

Micrographs of the fractured surfaces (Figure 10) reveal a more brittle behavior of the neat PLA, when compared to the two nanocomposites, which is in agreement with the stress–strain graphs produced during the experiments of this work. The more ductile behavior of the nanocomposite is also verified by the micrographs taken by the fractured areas of the specimens. In addition, it was evident that the extrusion process produced nanocomposites with no internal structuring defects, since a uniform structure can be observed in all different micrographs of materials developed in this work.

### 4.6. AFM Microstructural Analysis

Regarding the AFM data results in Figure 11, the SiO_2_ filler addition on the PLA polymer matrix seems to increase the surface roughness of the produced filaments, while no significant changes in the filament roughness (root–mean–square roughness–Rq and roughness average–Ra) was observed between the different filler concentrations.

Filaments from all the materials developed in the study (pure PLA and PLA/SiO_2_ nanocomposites with different concentrations) were found to contain similar grooves on the side surface that AFM is incapable of accurately measuring. Data results measured from the smoother part of the filaments’ side surfaces showed that there was a slight increase of Rz with the increase of the filler concentration.

## 5. Conclusions

In this study, novel nanocomposite filaments comprising of PLA/SiO_2_ were developed in various concentrations for application in FFF 3D printing technology, an approach not presented in the literature so far in terms of the nano fillers used with this specific polymer matrix and the methodology for the implementation of the study.

**Figure 12:**
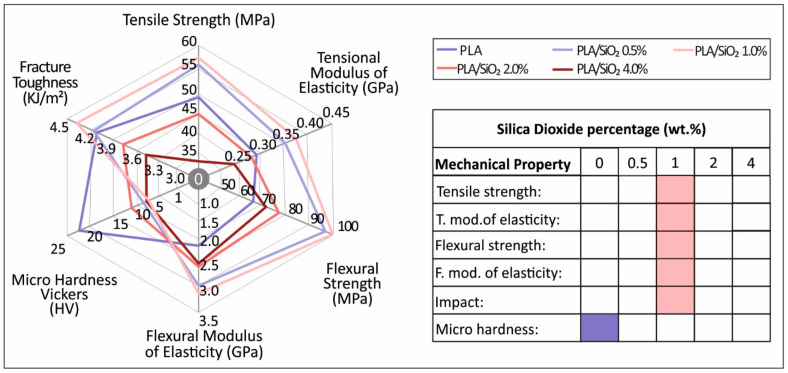
Summary of the mechanical properties results for all materials tested in this study. Highest values measured at each test is marked to the filler percentage appeared.

The overall results of this study are depicted in Figure 12. It was shown that the addition of SiO_2_ nanofiller in powder form to the PLA polymer matrix overall increases the mechanical properties of the polymer, with the highest increase recorded at 1.0 wt.% concentration. As the filler concentration increases for concentrations higher than 1.0 wt.%, the mechanical strength decreases. On the other hand, at the highest concentration prepared for this work of 4.0 wt.% the nanocomposite showed mild antibacterial properties, which were more effective against the *S. aureus*, making it a promising material for various 3D printing applications that require antibacterial properties.

Such nanocomposite materials having physically and mechanically improved properties can be easily fabricated for direct commercial or industrial implementation, while incorporating a viable, clean (solvent-free) and commercially applicable methodology.

The results and the outcome of this work will trigger further research endeavors in the direction of optimizing the process for direct industrial use and the study of the material at higher filler concentrations to determine the effect of the higher concentrations on its mechanical and its antibacterial properties. Additionally, the antibacterial process followed could be seen as a screening process and further studies according to the corresponding antibacterial standards and protocols need to be conducted, in order to more reliably determine the effect of the filler on the antibacterial activity of the PLA/SiO_2_ nanocomposites.

## Data Availability

The data presented in this study are available on request from the corresponding author.
